# Tools to analyze the organization and formation of the germline cyst in zebrafish oogenesis

**DOI:** 10.1242/dev.201349

**Published:** 2023-06-30

**Authors:** Vineet Kumar, Yaniv M. Elkouby

**Affiliations:** ^1^ Department of Developmental Biology and Cancer Research, The Hebrew University of Jerusalem Faculty of Medicine, Ein Kerem Campus, Jerusalem 9112102, Israel; ^2^ Institute for Medical Research – Israel-Canada (IMRIC), Ein Kerem Campus, Jerusalem 9112102, Israel

**Keywords:** 3D deep-learning data, Germ cell development and morphogenesis, Live manipulation by laser-ablation, Microscopy image analysis, Oogenesis, Germline cyst, Zebrafish ovary

## Abstract

Oocytes develop in the germline cyst, a cellular organization in which germ cells are tightly interconnected and surrounded by somatic cells. The cyst produces oocytes for follicle formation and is a hub for essential processes in meiosis and oocyte differentiation. However, the formation and organization of the cyst, and their contribution to oocyte production in vertebrates remain unclear. Here, we provide tools for three-dimensional and functional *in vivo* analyses of the germline cyst in the zebrafish ovary. We describe the use of serial block-face scanning electron microscopy (SBF-SEM) to resolve the three-dimensional architecture of cells and organelles in the cyst at ultrastructural resolution. We present a deep learning-based pipeline for high-throughput quantitative analysis of three-dimensional confocal datasets of cysts *in vivo*. We provide a method for laser ablation of cellular components for manipulating cyst cells in ovaries. These methods will facilitate the investigation of the cyst cellular organization, expand the toolkit for the study of the zebrafish ovary, and advance our understanding of female developmental reproduction. They could also be further applied to the investigation of other developmental systems.

## Teaser

**Summary:** A description of a set of imaging-based tools for automated 3D analyses and functional live manipulations *in vivo* of the germline cyst, a cellular hub for oocyte production, in the zebrafish ovary.

## INTRODUCTION

**Fig. 1.**
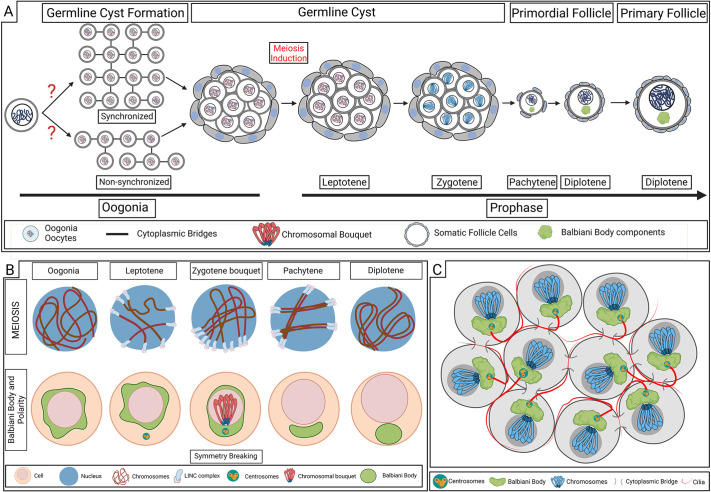
**The germline cyst.** (A) The germline cyst in early oogenesis. The number, pattern and synchrony of oogonial divisions that construct the cyst are unknown. The first stages of meiotic prophase (leptotene, zygotene) are executed in the cyst. The oocyte leaves the cyst to form the primordial follicle by the pachytene stage, and arrests at diplotene in the growing primary follicle. (B) Major processes in meiotic chromosomal pairing and oocyte polarity are executed in the cyst. Top: Nuclear (blue circle) dynamics of chromosomal (dark red) pairing, in which telomeres are loaded on the nuclear envelope (NE) at the leptotene stage and associate with perinuclear microtubules (not depicted) via Sun/KASH (LINC) complexes on the NE and the Dynein motor protein. Telomere movement on the NE (sliding on perinuclear microtubules) shuffles chromosomes, driving their homology searches. Telomeres ultimately cluster on the NE apposing the centrosome, forming the chromosomal bouquet configuration, which contributes to chromosomal pairing while oocytes develop in the cyst. In the follicle, paired chromosomes remain associated via chiasmata through pachytene and diplotene stages. Bottom: Dynamics of oocyte polarity and Balbiani body (Bb) formation. In the cyst, Bb components (green) are randomly distributed in oogonia and polarize for the first time around the centrosome and apposing the telomere (blue) cluster of the bouquet during symmetry breaking at zygotene stages. In the follicle, polarized Bb components form the mature Bb. (C) Schematic of a germline cyst of oocytes at the zygotene stage, which execute the chromosomal bouquet and symmetry-breaking events; oocytes are connected by cytoplasmic bridges (CBs) and form the zygotene cilia (red), which extend between them. Centrosomes are localized adjacent to CBs of the last division. Created with BioRender.com.

Oogenesis is a dynamic process that is essential for sexual reproduction. From insects to mammals, early oocytes develop in a cellular organization called the germline cyst, in which germ cells are clustered, interconnected, and collectively enveloped by somatic cells (Niu and Spradling, 2022). The germline cyst is formed by oocyte mitotic precursor cells called oogonia (Fig. 1A,C). Oogonia undergo several mitotic divisions with incomplete cytokinesis (Fig. 1A), which retains cytoplasmic bridges (CBs) with stabilized midbodies between daughter cells (Marlow and Mullins, 2008; Leu and Draper, 2010; Elkouby et al., 2016; Mytlis et al., 2022; Greenbaum et al., 2009). Oogonial incomplete cytokinesis results in cysts that comprise interconnected germ cells. Oocyte differentiation begins with entry into meiosis within the germline cyst, and, in zebrafish and mice, oocytes continue to develop in the cyst until they leave it to form the primordial follicle by the pachytene stage of meiosis (reviewed by Elkouby and Mullins, 2017a) (Fig. 1A).

The germline cyst serves as a hub for key events in oogenesis. Crucial events in meiotic prophase, including the induction of double-strand breaks and chromosomal pairing, occur in the cyst (Elkouby and Mullins, 2017a) (Fig. 1B, top; see legend for details). A direct connection between the meiosis program and the morphological organization of the cyst was unraveled with our recent identification of the zygotene cilium, an oocyte primary cilium that forms specifically in the germline cyst of zebrafish and mice (Mytlis et al., 2022, 2023) (Fig. 1C). Meiotic chromosomal pairing is mechanically controlled by perinuclear microtubules that grow from the centrosome microtubule organizing center (MTOC) (reviewed by Rubin et al., 2020; Kim et al., 2022; Burke, 2018) (see also Fig. 1B). Zygotene cilia connect to the oocyte MTOC machinery and extend extracellularly between oocytes in the cyst (Mytlis et al., 2022) (Fig. 1C). Loss of the zygotene cilium in zebrafish results in defected and delayed prophase, as well as in cyst disintegration, and consequently leads to ovarian dysgenesis and deficient fertility (Mytlis et al., 2022).

In addition to meiosis, the formation of a conserved oocyte organelle, called the Balbiani body (Bb) (Escobar-Aguirre et al., 2017b) begins in the cyst (Elkouby et al., 2016) (Fig. 1). The Bb is essential for oocyte polarity and embryonic development in zebrafish (Marlow and Mullins, 2008; Escobar-Aguirre et al., 2017b) and is associated with primordial follicle formation in mice (Lei and Spradling, 2016). In zebrafish, Bb formation is initiated in the cyst when the centrosome MTOC breaks the oocyte symmetry during the zygotene stage in prophase (Elkouby et al., 2016) (Fig. 1B, bottom). Evidence suggests that similar mechanisms initiate Bb formation in the cyst in mammals and insects (Tworzydło et al., 2016; Lei and Spradling, 2016). Upstream of symmetry breaking in zebrafish, the last mitotic division in the oogonial cyst has been proposed to position the centrosome and align polarization, as during symmetry breaking the centrosome localized adjacent to the CB (Elkouby et al., 2016) (Fig. 1C). These observations suggest a functional link between cyst organization and oocyte polarity (Elkouby et al., 2016). Altogether, major processes in oogenesis, including meiosis, Bb formation and oocyte polarity, emphasize potential roles for cyst organization (Fig. 1C), demonstrating the need for a better understanding of this cellular hub.

Most of our current understanding of the cyst is derived from the *Drosophila* model. In *Drosophila* ovaries, oogonia undergo exactly four rounds of mitotic divisions, forming a cyst of 16 cells (Hinnant et al., 2020). *Drosophila* cyst divisions are synchronous and generate orderly, organized cysts with predictable connections between sister cells (Nashchekin et al., 2021). In the *Drosophila* cyst, only one cell is specified as the oocyte and the remaining 15 function as supporting nurse cells that deliver material through CBs to the oocyte, in a process called dumping (Lu et al., 2017; Quinlan, 2016). Interestingly, a similar dumping mechanism, whereby nurse-like cells transfer material to a presumptive oocyte, was recently reported in mice (Niu and Spradling, 2022; Lei and Spradling, 2016). However, a variety of cyst organizations exist in nature. The structure of cysts can be represented using cell lineage trees (CLTs), where each cell and CB are defined as edge and node of the tree, respectively (Koch and King, 1969; Gondos et al., 1971; Haglund et al., 2011). Varying patterns of cell divisions in different species generate CLTs of distinct sizes and topology.

CLT networks can be categorized in five primary classes (Świątek and Urbisz, 2019; Diegmiller et al., 2022). In the two-cell network class, an oocyte is connected to a single support cell (termed nurse cell) that transports material to the oocyte, and this class is found in annelid worms (Brubacher and Huebner, 2009), the biting midge (Wang et al., 2020), earwigs (Yamauchi and Yoshitake, 1982; Tworzydło and Kisiel, 2010; Tworzydło et al., 2010) and multiple fungus gnats (Berry, 1941; Gutzeit, 1985). In the bilinear chain networks class, cysts are composed of two long strips of support cells emanating from centrally placed oocyte. Such cysts are formed in springtails (class Entognatha) (Matsuzaki, 1973; Biliński, 1983, 1993), in polychaetous annelid plumed worms (Anderson and Huebner, 1968), the springtime fairy shrimp (Kubrakiewicz et al., 1991) and net-winged insects (order Neuroptera) (Kubrakiewicz, 1997).

More complex classes are common. In the cytophore ring networks class, cysts are composed of a ring of cells surrounding a central anucleated cell called a cytophore (Świątek et al., 2009, 2018, 2020; Urbisz et al., 2017). In some ring networks, one of the peripheral cells of the ring becomes the oocyte, whereas the rest become nurse cells. In others, multiple oocytes, develop within a single cyst, as in *Piscicola geometra* (Spałek-Wołczyńska et al., 2007; Świątek et al., 2009). In the 2n branched networks class, cysts are formed as a result of synchronous cell divisions, forming a symmetrical structure, which at each division step comprises 2n cells, and where the two most central cells are connected to an equal number of cells. Examples of varying numbers exist. These include 4-cell cysts (*n*=2 in the scorpion fly; Ramamurty, 1967), 8-cell cysts [*n*=3 in whirligig beetles (Matuszewski and Hoser, 1975), *Dineutus nigrior* (Hegner and Russell, 1916) and the majority of moths and butterflies (Yamauchi and Yoshitake, 1984; Marec et al., 1993)], 16-cell cysts [*n*=4, e.g. in the oriental fruit fly (Lee, 1985), winter crane flies (Mazurkiewicz and Kubrakiewicz, 2005) and *Drosophila*], and 32-cell cysts [*n*=5, e.g. in the mole flea (Büning and Sohst, 1988) and parasitic wasp (Eastin et al., 2020)].

The last class of cysts is asymmetric networks (Diegmiller et al., 2022), which form by nonsynchronous cell division. For example, the net-spinning caddisfly forms a 3-cell cyst with an oocyte at one end (Matsuzaki, 1972), and the green lacewing forms a 12-cell cyst (Rousset, 1978). Another category of an asymmetric network is found in *Linepithema humile* and the bumblebee *Bombus terrestris*, which form tree-like cysts with numerous long linear branches (Eastin et al., 2020) and do not fit well within any of the class categories.

Considering this high variability of cyst organizations and despite vast mechanistic knowledge from *Drosophila*, the formation and organization of the cyst in vertebrates, including mammals, is poorly understood. The number of cells in the vertebrate cyst is uncertain. In the mouse, cysts were reported to contain an average of 30 cells (Lei and Spradling, 2013), and cysts break down followed by the formation of clonally unrelated clusters from cyst cells (Lei and Spradling, 2013). In *Xenopus*, cysts contain up to 16 cells (Kloc et al., 2004), whereas medaka cysts contain up to 30 cells (Nakamura et al., 2010), and in zebrafish the definitive number of cells in the cyst is unknown. In humans, oogonial cells have been described to be predominantly found in groups (Kurilo, 1981) or nests (Anderson et al., 2007) in fetal ovaries. These nests likely represent cysts, or, alternatively, nests of smaller clonally unrelated cysts, as was shown in mice (Lei and Spradling, 2013). However, whether they are connected by CBs and the number of cells per nest are unclear. Overall, whether oogonial divisions that construct the cyst are synchronized, their pattern of divisions (Fig. 1A) and the function of the cyst in vertebrate oogenesis are unknown.

In zebrafish, the cellular processes of oogenesis and ovarian development are executed and genetically regulated similarly to mammals (reviewed by Elkouby and Mullins, 2017a; Li and Ge, 2020). Owing to multiple experimental advantages, the zebrafish ovary is as an excellent model for the study of oogenesis (Elkouby and Mullins, 2017b; Li and Ge, 2020), and the toolbox for the investigation of zebrafish oogenesis has been significantly expanded. Advances in genetics (Jamieson-Lucy et al., 2022; Leerberg et al., 2019; Beer and Draper, 2013), quantitative and live ovarian imaging (Mytlis et al., 2022; Mytlis and Elkouby, 2021), live manipulations of cultured ovaries (Deis and Elkouby, 2022 preprint), and various proteomic and genomic approaches (Jamieson-Lucy et al., 2022; Liu et al., 2022; Bogoch et al., 2022) have impacted the field greatly. Nevertheless, a fundamental understanding of the cyst is still needed. Direct investigation of the germline cyst has been challenged by two technical issues: (1) the thick sample size of the ovary, which restricts the penetration of probes, limiting analyses deep in the tissue, and (2) the limitation of available image-processing and automatic-segmentation tools for distinguishing between germline cyst cells and their closely surrounding somatic cells.

Here, we provide methodologies and step-by-step protocols for quantitative three-dimensional analyses of the germline cyst in high throughput and *in vivo*. We describe the use of serial block-face scanning electron microscopy (SBF-SEM) in ovaries and provide methods for segmenting and rendering SBF-SEM data to characterize the spatial organization of the germline cyst in 3D and at ultrastructural resolution. We present the implementation of the deep-learning algorithms StarDist (Weigert et al., 2020; Schmidt et al., 2018) and Cellpose (Stringer et al., 2021) for the segmentation and analysis of entire germline cysts from 3D ovary image datasets. Finally, we present a methodology to manipulate cyst cells for functional investigation by laser-induced ablation of cellular components of interest, using multiphoton microscopy. Altogether, these methodologies will facilitate the systematic and timely investigation of the cyst cellular organization in zebrafish oogenesis, and could be directly transferred for the investigation of many developmental systems.

## RESULTS

### Manual segmentation of SBF-SEM in TrackEM2

The state-of-the-art SBF-SEM technique provides a combination of conventional scanning electron microscopy (SEM) with three-dimensional image acquisition. In this setup, the sample is embedded in a block and after each scan, a microtome scrapes off a 70 nm-thick surface layer of the sample, exposing a new surface for imaging. From multiple iterations of image acquisition and sectioning cycles in a region of interest, 3D images at electron microscopic resolution are constructed.

**Fig. 2.**
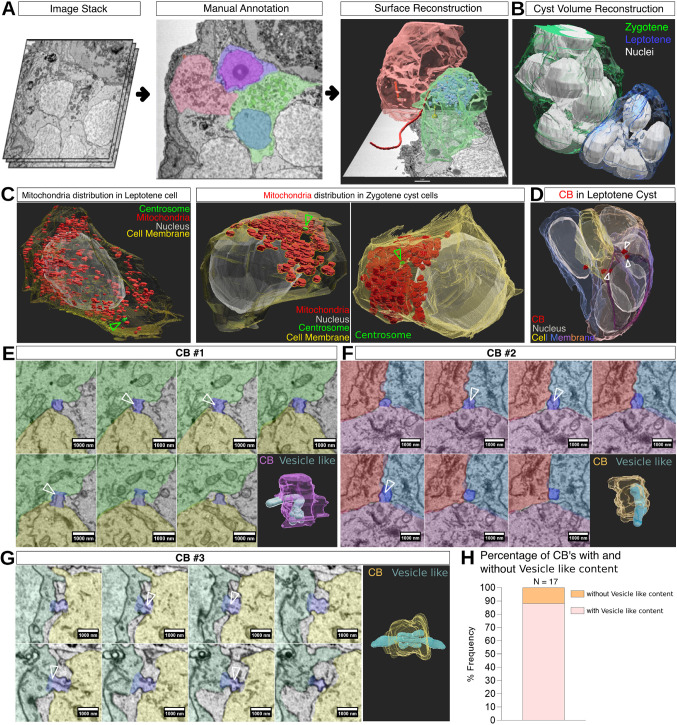
**Three-dimensional reconstruction of the germline cyst from ovary SBF-SEM image datasets.** (A) Selected steps from the segmentation protocol. Stacks of images are combined (left), and cellular features of interest are manually annotated (middle), followed by surface and volume reconstruction (right), showing: the zygotene cilium (maroon), mitochondria (blue) and cytoplasmic membranes (pink and green). Cells in the middle and right panels are color coded to match. See Movie 1. (B) A general zoomed-out image of volume reconstruction of two adjacent leptotene (blue) and zygotene (green) cysts. (C) Detection of organelle distribution. SBF-SEM segmentation detects mitochondria distribution during oocyte polarization dynamics. Before symmetry breaking (left; leptotene), mitochondria are randomly distributed in the cytoplasm and not specifically enriched adjacent to the centrosome (arrowhead). In contrast, during symmetry breaking at the zygotene stage (two examples are shown: middle, right), mitochondria are polarized adjacent to the oocyte centrosome (arrowheads). (D) Detection of CBs in a cyst. Arrowheads point to three CBs that are connected to a single oocyte. See Movie 2. (E-G) CB morphology. Three representative CBs are shown. For each CB, a montage of sequential section images visualizes the CB (purple), as well as its connected and surrounding cells (segmented in different colors). Vesicle-like material is detected in sequential sections through the CB (arrowheads). 3D reconstruction (bottom-right panels) confirms that the vesicle-like material is found within and extends through the CB. Scale bars: 1 µm. (H) Frequency of CBs with and without vesicle-like content (*n*=17 CBs).

We implemented SBF-SEM to characterize the cellular organization of the germline cyst. 3D datasets of cyst images acquired at electron microscopic resolution were processed following our detailed protocol (see Materials and Methods) using TrakEM2 software (Cardona et al., 2012) (Fig. 2). We captured three cysts in two regions of interest (ROIs) from two ovaries, each spanning over 30 h of image acquisition. We segmented various cellular components in cysts of leptotene- and zygotene-stage oocytes, including nuclei, cell membranes, CBs, centrosomes and zygotene cilia (Mytlis et al., 2022) (*n*=30 cells from three cysts and two ovaries). Segmented labels were then used to generate volume rendering of each component of the germline cyst (Fig. 2A,B). From the generated volume renders, we resolved and visualized cell–cell connections, the morphology of cyst cells, and ciliary extension through the cyst, in 3D (Mytlis et al., 2022) (Fig. 2).

We demonstrate here that our SBF-SEM segmentation can also be applied for resolving subcellular organelles in ovaries, including mitochondria, as well as their cellular distribution (Fig. 2C; Movie 1). We previously identified a symmetry-breaking event in the oocyte at the zygotene stage (Elkouby et al., 2016). As detected by confocal and two-dimensional transmission electron microscopy (TEM) images, Bb components, including mitochondria, polarize adjacent to the oocyte centrosome at zygotene stages, but are dispersed randomly at earlier stages (Elkouby et al., 2016). We validated the precise detection of organelles in our pipeline by testing whether it can capture these mitochondria dynamics. Using our pipeline to segment mitochondria and centrosomes, we detected clusters of localized mitochondria adjacent to the oocyte centrosomes at the zygotene stage (*n*=24 oocytes; Fig. 2C, right panels), whereas mitochondria in leptotene-stage oocytes appeared dispersed and not specifically enriched adjacent to centrosomes (*n*=8 oocytes; Fig. 2C, left). Our SBF-SEM segmentation thus reliably detects organelles in ovaries and confirms their cellular and developmental dynamics during oocyte polarization in 3D.

Next, we detected intricate and fine cellular and subcellular morphology with potential direct functional relevance. Considering that CBs define the cyst organization, we examined their morphology. Examining CBs by confocal analyses requires specific antibodies for CB components that are not easily available in zebrafish, or generation of transgenic lines. Attempting to analyze CBs in thin sections by TEM is challenging because this method lacks the 3D data to detect entire oocytes or CBs. These challenges are overcome by our SBF-SEM pipeline, and we were able to detect CBs through cysts (Fig. 2D-G).

We were previously able to reliably measure the size of the CB using SBF-SEM, resulting in an average diameter of 567±172 nm (Mytlis et al., 2022). We therefore went on to investigate how many CBs can be detected per oocyte, which can indicate the number of previous divisions, and is unknown in zebrafish. An example for 3D CB detection in a cyst is shown in Fig. 2D and Movie 2. Although most oocytes were detected with one or two CBs (*n*=22 cells in three cysts from two ovaries), in two cysts we detected one oocyte with three CBs per cyst (Fig. 2D; Movie 2), indicating that at least one cell in the cyst can undergo three rounds of divisions. Although, typically for SBF-SEM, this analysis is limited to a small sample size (see Discussion), a scenario wherein most oocytes in a cyst contain one or two CBs, and few contain three, best fits the branched network class of cysts, but this remains to be determined. Whether the zebrafish cyst forms by synchronous or asynchronous divisions needs to be addressed by live time-lapse imaging. Nonetheless, these data provide the first indication of the organization of the zebrafish germline cyst as a branched network.

An important feature of the cyst is inter-communication between cells through the CB connections of their cytoplasm. The dumping mechanism in *Drosophila* transfers material, including mRNA and proteins, from nurse cells to the oocyte through CB ring canals (Quinlan, 2016; Lu et al., 2017) and a similar mechanism was recently proposed in mice (Lei and Spradling, 2016; Niu and Spradling, 2022). However, whether material is transferred between cyst cells in zebrafish is unknown. We therefore examined the content of CBs in our dataset.

Capturing the entire volume of CBs, we could detect vesicle-like structures in the vicinity of the CBs or in their opening. Fig. 2E-G shows three representative examples of CBs that contain vesicle-like material, as shown by montage images of serial sections, as well as by their 3D segmentation generated by our pipeline. As demonstrated in the 3D segmentation, the vesicle-like material is clearly visible in the CB vicinity and extends into one or both connected oocytes. We found that 88% of CBs contained vesicle-like structures (*n*=17; Fig. 2H), suggesting that such vesicle-like structures in CBs are common. These presumptive vesicles were detected in consecutive sections through the CB (montage images in Fig. 2E-G), but did not encompass the entire CB diameter (Fig. 2E-G), which could explain why they have been overlooked in 2D TEM analyses. Although they need to be confirmed by live time-lapse imaging, these observations suggest, for the first time in zebrafish, the transfer of material between cyst cells, which could be key for various aspects of oocyte development and/or cyst regulation.

Thus, SBF-SEM and our segmentation pipeline provide a powerful method for analyzing 3D cellular organizations at ultrastructural resolution to decipher sub- and intercellular structures of interest comprehensively and accurately. This is a promising approach for identifying previously unknown cellular features, as we show here and have shown previously for the zygotene cilium (Mytlis et al., 2022). Generated label images can be further used in ImageJ for quantitative analysis of the physical parameters of cells, including volume, surface area and additional parameters of interest.

A potential limit of SBF-SEM is difficulties in analyzing a large number of ovaries per sample. SBF-SEM requires a long image acquisition, and it can be demanding to identify the correct and complete ROI in the whole tissue, which might require several acquisition attempts. However, once identified and characterized, the novel and unequivocally precise data extracted from SBF-SEM can be studied by more robust imaging approaches such as confocal microscopy. To complement SBF-SEM for the robust analysis of cysts in 3D based on confocal microscopy, we developed a deep-learning based approach, as described below.

### Deep learning-assisted instance segmentation of nuclei and cells

We established a method for robust analyses of cysts from 3D confocal microscopy datasets. The developing ovary is a complex organ, which contains a variety of cell types, including somatic cells, germline stem cells, mitotic oogonia and differentiating oocytes at different stages. Furthermore, differentiating oocytes in developing ovaries at these stages range widely in sizes, from oocyte-precursor oogonia, which are 9-11 µm in diameter (Elkouby and Mullins, 2017b; Elkouby et al., 2016), to typically up to ∼70 µm oocytes in primary follicles (Elkouby and Mullins, 2017b; Elkouby et al., 2016; Kobayashi et al., 2021). The complexity increases when the size of somatic cells is taken into consideration. Such cellular complexity challenges the application of automatic segmentation algorithms in images, resulting in misidentification of cells and nuclei.

**Fig. 3.**
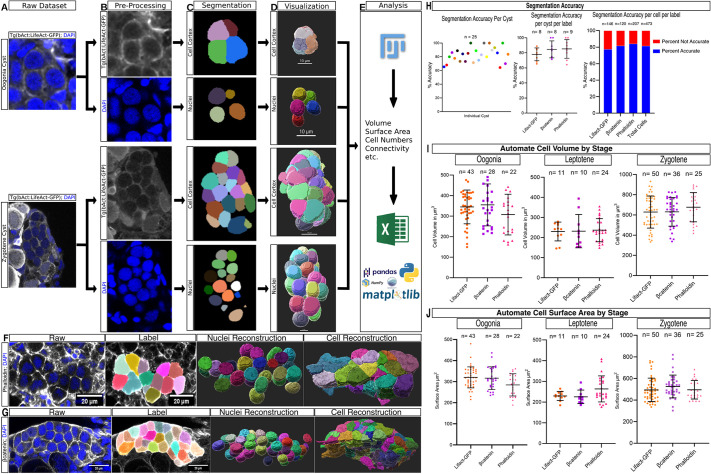
**Deep learning-assisted instance segmentation of the germline cyst in 3D.** (A,B) Raw images of cysts (A) are pre-processed in Fiji for brightness/contrast adjustments (B). Cysts of two stages are shown, oogonia (top), and zygotene (bottom), expressing transgenic Lifeact-GFP [*Tg(βact:LifeAct-GFP)*; actin on cell cortex, gray] and counterstained with DAPI (blue). The protocol is suitable for other cyst stages and labels for cellular markers of interest. (C) Segmentation: single-frame representation of instance segmentations generated by Cellpose (cell cortex, top for each cyst) and StarDist (nuclei, bottom for each cyst). (D) Visualization: three-dimensional volume reconstructions of data from C in Imaris. (E) Analysis: labels generated in C are processed in Fiji for quantification of physical properties, such as volume, surface area etc., for statistical analysis in Excel or Python-based data-processing pipelines. See Movies 3 and 4 for the steps shown in A-E. (F,G) Application of the protocol on cysts from ovaries labeled by the vital dye phalloidin (F; gray; actin on the cell cortex), or by antibody staining for β-Catenin (G; gray; adherens junctions on cytoplasmic membranes), and DAPI (blue). Panels are raw and labeled images (left), and 3D visualization of automatically segmented nuclei and cell cortex (right). (H) Plots showing the accuracy of automated segmentation of cells per cyst (left; each dot is a cyst); per cyst and per label from LifeAct, β-Catenin and phalloidin (middle); and per cell and per label (right), in which the right bar represents all cells pooled from all labels. (I,J) Sizes of segmented cells for oogonia, and leptotene and zygotene stages, as extracted from the automated segmentation as shown in E, by volume (I) and surface area (J). Note the consistent measures across all labels per stage. Error bars represent s.d.

We show here that cellular complexities in the juvenile ovary can be addressed and overcome robustly by using deep learning-assisted instance segmentation algorithms with custom models for cell types (Fig. 3; Movies 3, 4). Using this approach, we executed instance segmentation on high volumes of raw datasets in a robust and high-throughput manner (Fig. 3A-D), as detailed in our protocols (Materials and Methods). The generated labeled images can be utilized in various analysis pipelines (Fig. 3E). Here, we implement this approach on cysts of various sizes and developmental stages, including mitotic oogonia and meiotic leptotene-zygotene oocytes. The pipeline described in Fig. 3A-E shows examples of two types of cysts: a 4-cell cyst of oogonia (Fig. 3, top; Movie 3) and a 16-cell cyst of zygotene-stage oocytes (Fig. 3, bottom; Movie 4).

We show the 3D segmentation of nuclei and cell cortexes from a raw dataset of ovaries (Fig. 3A-E). Cell cortex structures were labeled and detected by three independent methods: (1) transgenic expression of Lifeact-GFP (cortical actin) (Fig. 3A), (2) phalloidin dye (cortical actin) (Fig. 3F), and (3) immunostaining using a β-Catenin antibody, which labels adherens junctions on the cortex of cyst oocytes (Elkouby et al., 2016) (Fig. 3G). In all cysts, nuclei were labeled and detected using DAPI (Fig. 3A-D; Movies 3, 4). We ran our pipeline on ovaries labeled with each of the above markers, and segmented cell borders and nuclei in 25 cysts of various stages from 13 ovaries.

Our segmentation detected individual cells in cysts with high accuracy. In some cases, because of the inevitable minimal variability of transgenic expression, dye detection or antibody staining, the labeling signal was weaker along a cell border. These specific cases of insufficient labeling quality resulted in the false merging of cells in a few optical sections. We tested the accuracy in our hands, and manually supervised the calling of cells in cysts and compared the automated calling to manual calling.

We calculated an accuracy rate for which the automated and manual callings were identical per cyst in data from all labels, as well as per cyst and per cell for each label (Fig. 3H). Accuracy rates were 77.3% of cells and 78% of cysts in the LifeAct-GFP-labeled ovaries (*n*=146 cells from eight cysts; Fig. 3H), 83.5% of cells and 85% of cysts in the phalloidin-labeled ovaries (*n*=207 cells from nine cysts; Fig. 3H), and 81% of cells and 82% of cysts in the β-Catenin-labeled ovaries (*n*=120 cells from eight cysts; Fig. 3H). Overall, we calculated 81% accuracy (*n*=473 cells from 25 cysts and 13 ovaries) of total cell calling from all cell border labels (Fig. 3H, right), which is consistent with the accuracy levels originally reported for Cellpose (Stringer et al., 2021). Cases of inaccurate calling in all labels can be very easily supervised by the user. The accuracy of DAPI-based nuclei calling was 78% (*n*=335 cells in 25 cysts from 13 ovaries), which is consistent with the accuracy originally reported for StarDist (Weigert et al., 2020; Schmidt et al., 2018). Thus, we were able to achieve optimal accuracy of labeling in whole ovaries. Additional training of these algorithms on ovary samples by further use is expected to increase their accuracy even further.

We next analyzed all accurately called cells. We extracted parameters for cell sizes by volume and surface area, per developmental stage. Oogonia cells showed consistent sizes, as labeled by all three markers, of approximately 307 µm^2^ surface area and 336 µm^3^ volume (Fig. 3I,J, left). Leptotene cells showed consistent sizes by all markers of approximately 249 µm^2^ surface area and 233 µm^3^ volume (Fig. 3I,J, middle). Finally, zygotene cells further showed consistent sizes by all markers of approximately 505 µm^2^ surface area and 645 µm^3^ volume (Fig. 3I,J, right).

The extracted characteristic volumes are more accurate than our manual oocyte staging criteria. We previously defined the characteristic size range of each stage, based on molecular markers (Elkouby et al., 2016; Elkouby and Mullins, 2017b; Mytlis et al., 2022). To systematically measure these diameters based on confocal imaging, we inevitably had to assume a spherical shape for oogonia and oocytes. However, oogonia and oocytes in cysts are not shaped as a perfect sphere and vary in morphology, as shown in our SBF-SEM and deep learning-based 3D segmentation pipelines (Figs 2, 3). Therefore, although our previous manual measures can still distinguish between developmental stages, the automated extracted values are much more accurate, and offer an improved tool for determining developmental stages. We conclude that our pipeline provides a robust, unbiased and precise tool for measuring cellular features of the cyst, and generate reliable data.

This powerful approach can be executed similarly on live-imaged ovaries, as well as on ovaries expressing or stained for various cellular markers of interest. Such experiments could be used to extract and analyze additional parameters of cyst cells in the future, such as, for instance, cell connectivity, by using the newly generated mCherry-Cep55l transgenic line, which labels midbodies (Mytlis et al., 2022). The developed pipeline is robust with many cysts from multiple ovaries being efficiently co-processed and analyzed. Importantly, it can also be extended to detect any cellular feature that can be visualized by confocal microscopy. The only requirement is that the analyzed 3D confocal images can be acquired in good quality, with sufficient signal/noise ratio that can be distinguished by the algorithms for proper segmentation. The segmentation of different features would also require specific training by the algorithm, but this is relatively straight-forward to perform.

### Laser-induced ablation of germline cyst cell organelles

Having established methodologies for characterizing cyst architecture, we next aimed to develop an imaging-based protocol for manipulating cyst cells for functional studies. In functional analysis of cells and cell compartments, addressing phenotypic dynamics at high temporal resolution by genetics can be limited. Laser-induced ablation allows the examination of immediate and highly dynamic phenotypes in real time. We developed a protocol to experimentally manipulate cellular components in live, whole-mount cultured ovaries while recording and analyzing the effects on cellular dynamics in real time. This protocol is based on and extends our protocols for live time-lapse imaging of cultured ovaries (Elkouby and Mullins, 2017b; Mytlis and Elkouby, 2021; Mytlis et al., 2022), adopting it to multiphoton microscopy (MPM). MPM offers deep penetration, reduced photodamage, minimal invasion over prolonged measurements, and fine laser precision for highly specific ablations.

**Fig. 4.**
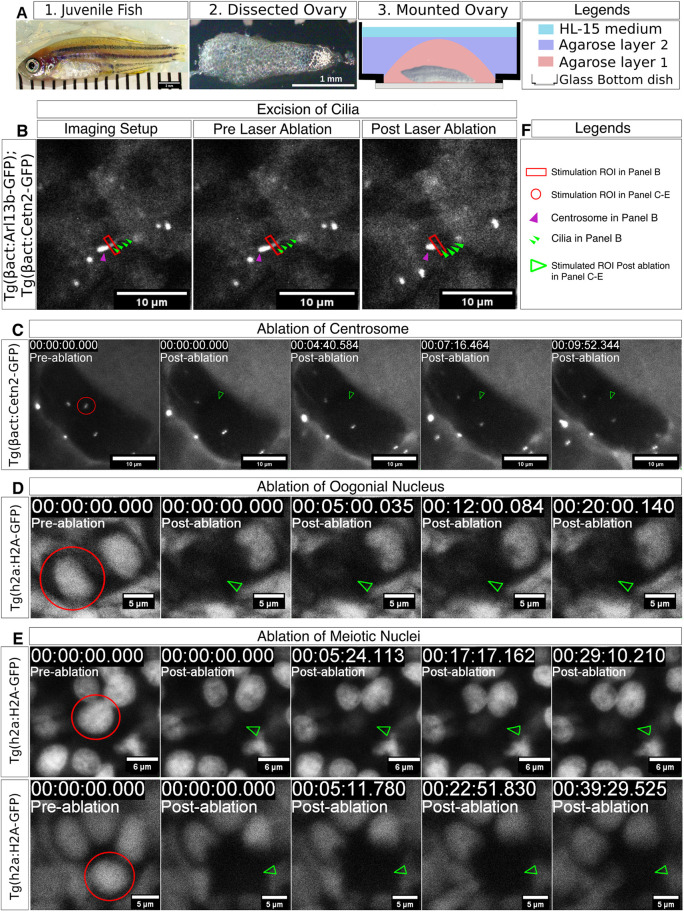
**Laser-induced ablation of cyst cell organelles.** (A) Ovary dissection and mounting: (1) A 6 wpf juvenile fish (SL=14.5 mm), from which an ovary is dissected (2) and mounted (3). (3) Ovary mounting setup: the ovary is mounted at the bottom of a glass-bottom dish inside two layers of HL-15 media containing low-melt agarose (pink and purple) and covered with HL-15 media (teal). (B) Ablation live time-lapse setup, showing the excision of the zygotene cilium in ovaries of a double-transgenic [*Tg(bact:Arl13b-GFP); Tg(bact:Cetn2-GFP)*] fish, labeling the centrosome (purple arrowhead) and cilia (green arrowheads). The laser ablation stimulation ROI is at the base of the cilium (red rectangle). The left panel shows the imaging setup before the beginning of the time-lapse experiment. The middle panel shows the image before ablation (−30 s). Ablation is executed for 30 s and the time at the end of ablation is defined as 00:00. The right panel shows the excised cilium at ∼13 s post-ablation. Scale bars: 10 µm. Images are snapshots from Movie 5. (C) Ablation of a single centrosome in a cyst using the *Tg(bact:Cetn2-GFP)* line and similar settings as in B. The selected centrosome (red circle ROI in the pre-ablated panel) is specifically ablated (green arrowhead in post-ablation panels), whereas the non-ablated control centrosomes remain intact. Images are sum-projection snapshots from Movie 6. Scale bars: 10 µm. (D,E) Ablation of single nuclei in cysts using the *Tg(h2a:H2A-GFP)* line and similar settings as in B, in a 2-cell oogonia cyst (D), as well as in meiotic leptotene-zygotene cysts with multiple cells (E). The selected nuclei (red circle ROI in pre-ablation panels) are ablated (green arrowheads in post-ablation panels), whereas the non-ablated control nuclei remain intact. Images are sum-projection snapshots from Movies 7-9. Scale bars: 6 µm (D); 5 µm (E). (F) A key for the features shown in B-E.

In this protocol (see Materials and Methods), cultured ovaries are mounted in 1% low-melt agarose in a glass-bottom culture dish and reinforced with another layer of low-melt agarose to minimize movements (Fig. 4A). On the microscope system, an ROI is located and marked (Fig. 4B, red rectangle). Live-imaging parameters were set to 60 s for pre-ablation recording, followed by stimulation for 30 s and then post-ablation recording up to 600 s. The incision laser power at the marked ROI for ablation was set to 2.0-8.0% out of a power source of ∼3 W.

Using this protocol, we previously manipulated the zygotene cilium in whole-mount cultured ovaries (Mytlis et al., 2022) (Fig. 4B). We used ovaries from a double transgenic line [*Tg(bact:Arl13b-GFP); Tg(bact:Cetn2-GFP)*], which simultaneously visualizes cilia and centrosomes (Borovina et al., 2010; Novorol et al., 2013). Upon laser-induced abscission of the zygotene cilium, we detected an immediate dislocation of its associated centrosome (Mytlis et al., 2022). Together with other experiments, this allowed us to conclude that the cilium acts to anchor the centrosome in the germline cyst (Mytlis et al., 2022). Thus, this protocol can address questions concerning fine cellular dynamics in real time within developing ovaries, where information from other approaches, such as genetics, can be limited.

Here, we demonstrate the successful application of our protocol to independently ablate three organelles in cysts within live cultured ovaries (Fig. 4B-E). First, we reproducibly demonstrate ciliary excision, and, second, we show the utility of the protocol in ablating the oocyte centrosome, as well as the nucleus. To excise the cilium (Fig. 4B; Movie 5), we used the double transgenic line *Tg(bact:Arl13b-GFP); Tg(bact:Cetn2-GFP)*, as described above. We marked an ablation ROI (Fig. 4B, red rectangle) at the base of the cilium (Fig. 4B, green arrowheads) and away from the centrosome (Fig. 4B, magenta arrowhead; Movie 5). Using the laser power parameters described above, we successfully excised the cilium (compare pre- and post-ablation panels in Fig. 4B and in Movie 5), without cellular damage (Movie 4) (Mytlis et al., 2022).

To ablate the oocyte centrosome (Fig. 4C; Movie 6), we used ovaries of the single transgenic line *Tg(bact:Cetn2-GFP)*, which visualizes centrioles. We imaged cysts pre-ablation, ablated a single centrosome (Fig. 4C, left, red circle; Movie 6) using same parameters as above, and monitored the cyst by subsequent time-lapse imaging for∼10 min, recording every ∼30 s (Fig. 4C; Movie 6). To confirm centrosome ablation and rule out its translocation to a different position, we imaged a stack of optical sections through the cyst *z-*axis as shown in the sum-projection images in Fig. 4C, which are snapshots from Movie 6. We were able to ablate the selected centrosome specifically, without affecting non-ablated control centrosomes (*n*=7 centrosomes in seven cysts, from four ovaries). Cysts appeared to remain vital post-ablation and during the course of recording, consistent with our previous experiments (Mytlis et al., 2022).

To ablate the oocyte nucleus (Fig. 4D,E; Movies 7-9), we used ovaries of the *Tg(h2a:H2A-GFP)* transgenic line, which labels Histone 2A, visualizing nuclei in ovaries. Using the same settings, we ablated a single nucleus (Fig. 4D,E, left, red circle; Movies 7-9) in cysts, and monitored them by time-lapse imaging, acquiring *z*-stacks of cyst images every ∼60 s for 20-40 min (Fig. 4D,E; Movies 7-9). We were able to ablate a single nucleus specifically, whereas the non-ablated control nuclei were unaffected and cysts appeared to remain vital post-ablation and during the course of imaging (*n*=10 nuclei in ten cysts, from seven ovaries). Fig. 4D,E and Movies 7-9 show examples of successful ablations of single nuclei in a 2-cell oogonia cyst (Fig. 4D; Movie 7), as well as in meiotic leptotene-zygotene cysts (Fig. 4E; Movies 8, 9), demonstrating consistent results in cysts of different cell number and stage.

These experiments demonstrate that our ablation protocol is suitable for ablating different organelles of various cellular and extracellular positions in the cyst (cytoplasmic centrosome versus the cell-protruding cilium), as well as sizes (centrosome versus nucleus). This versatility suggests that this protocol can be extended to be efficiently performed in ovaries expressing other transgenic reporters or labeled by vital dyes of interest, for manipulation of other organelles. Our method could thus be utilized to manipulate various cellular components of interest, paving the way for numerous possibilities for analyzing cellular developmental dynamics in real time.

## DISCUSSION

From insects to mammals, key events in oogenesis are executed in the context of the germline cyst. Deciphering the cellular architecture and functions of the cyst is required for understanding the developmental mechanisms that underlie female reproduction, but these remain unclear in vertebrates. We report powerful protocols for understanding the spatial-temporal development and 3D morphology of the germline cyst in zebrafish, and provide them in reproducible step-by-step protocols.

### Tools for analyzing the germline cyst

Our protocol for manual segmentation of SBF-SEM in TrakEM2 outlines volume reconstruction from an SBF-SEM dataset to visualize the germline cyst (Fig. 2; Movies 1, 2). Our protocol utilizes ultrastructural resolution images for generating 3D views of the morphology of the germline cyst, including the intercellular organization of cyst cells and their subcellular structures. This tool provides valuable information for deciphering the cyst functional architecture and dynamics, as we show for the zygotene cilia, for mitochondria subcellular distribution during oocyte polarization dynamics (Fig. 2C; Movie 1), and for CB morphology, number (Fig. 2D; Movie 2) and content (Fig. 2E-H).

SBF-SEM is extremely powerful in providing accurate 3D information, which is valuable for unequivocally characterizing and validating features of interest, and for discovering others that have been overlooked by other approaches. However, SBF-SEM can be limiting in the number of samples that can be analyzed. Sequential, iterative SEM imaging and sectioning at high resolution results in prolonged image acquisition (>30 h in our hands). It can be demanding to identify the ROI deep in tissues while keeping it intact for imaging, and there is a trade-off between the *xy* resolution and the ROI size. Notably, in cases in which ROIs are adjacent, we were able to maximize a single imaging session to acquire more than one ROI. We recommend combining SBF-SEM with more robust imaging approaches, such as confocal microscopy, to enable analyses of a high number of samples during functional studies.

Our protocol for deep learning-assisted instance segmentation outlines its implementation for automated detection, labeling and analysis of germline cyst cells and nuclei from ovary 3D image datasets in a high-throughput manner (Fig. 3; Movies 3, 4). Automatically analyzed datasets provide unbiased characteristic features of cyst cells, including their physical parameters, such as volume and surface area, and how they change during development. Applying this tool to datasets in which additional cellular structures are visualized by transgenic reporter lines, vital dyes, or immunostaining will provide ample information and an unprecedented quantitative understanding of the germline cyst. For example, visualizing the midbody in CBs in a Cep55l transgenic line (Mytlis et al., 2022) will reveal the network of cellular connections in the cyst, from which it will be possible to extract the patterns of divisions that construct the cyst.

This approach can be used to detect any cellular feature that can be visualized by confocal microscopy, given that images are acquired at sufficient quality and by uniform settings as appropriate for quantitative imaging (detailed for ovaries by Elkouby and Mullins, 2017a,b). Analyses of different cellular features would require specific training by the algorithm, which is feasible on conventional computers. In our work, we have reached optimal accuracy for automated identification of cell borders, as labeled by several markers, as well as of nuclei (Fig. 3), which is consistent with the accuracy originally reported for these algorithms (Schmidt et al., 2018; Weigert et al., 2020; Stringer et al., 2021). We recommend that users will manually supervise the automated analyses to determine the detection accuracy in their systems.

Our protocol for laser-induced ablation of organelles in germline cyst cells outlines a method for laser-assisted ablation of subcellular features in the germline cyst (Fig. 4; Movies 5-9). This protocol enables the experimental manipulation of cellular components of interest to study their real-time effects on cyst cell development over time. Genetic approaches and data from fixed samples can be limited in providing direct and precise functional information in real time and at high resolution. Even sophisticated conditional genetics resulting in tightly controlled mutant or loss-of-function conditions and combined with live imaging can be limited in providing information on rapid and highly dynamic phenotypes. Our protocol for laser ablation of cellular structures of interest in the zebrafish ovary comprises a complimentary approach to provide exactly this type of information and offers a new tool for functional analyses in the germline cyst.

We demonstrate that this protocol is useful for manipulating cellular organelles of various positions and sizes in cyst cells (Fig. 4B-E; Movies 5-9). The only requirements for this methodology are the use of a multiphoton microscope and the availability of a transgenic line that visualizes cellular features of interest for live imaging. To transfer our live-culturing protocol of ovaries from confocal settings to imaging by multiphoton microscopy, we only needed to minimally adjust our mounting set-up. When applying our protocol in other systems, we recommend that users carefully adjust the settings of the ablating laser stimulation according to the tissue, laser wavelength and ROI. Our reported settings provide an excellent reference point.

### The germline cyst and developmental reproduction in zebrafish and humans

The germline cyst is a hub for the production of primordial follicles. Oocytes begin their differentiation and develop in the cyst, from which they are subsequently released to form primordial follicles. Major processes in oocyte differentiation take place in the cyst. For example, the essential events of meiotic prophase, including chromosomal pairing and homologous recombination, are executed in the cyst. Importantly, aneuploidy in human eggs is a major cause for miscarriages and infertility, but the mechanistic defects are unknown because we lack a complete understanding of these early stages (Nagaoka et al., 2012; MacLennan et al., 2015; Webster and Schuh, 2017). A direct connection between cyst morphogenesis and chromosomal pairing regulation was revealed by the identification of the zygotene cilium in zebrafish and mice (Mytlis et al., 2022, 2023). Thus, characterizing cyst architecture and formation, and deciphering its regulatory mechanisms is essential for understanding meiotic prophase mechanisms.

In humans, the corresponding stages of oogenesis occur in the developing fetal ovary. At around gestational weeks 10-11, oogonia are predominantly found in groups or nests (Kurilo, 1981; Anderson et al., 2007; Farini and De Felici, 2022), which likely represent cysts, or nests of clonally unrelated smaller cysts, as was shown in mice (Lei and Spradling, 2013). During gestational weeks 14-26, meiosis is initiated non-synchronously, and mitotic oogonia differentiate and give rise to primordial and primary follicles (Kurilo, 1981; Farini and De Felici, 2022). By weeks 35-40, most oocytes are found in primary follicles (Kurilo, 1981; Farini and De Felici, 2022). Strikingly, ∼80% of the initial germ cell pool is cleared by apoptosis before and slightly after birth (Hunter, 2017). A similar clearance of ∼80% of the initial pool also occurs around these stages in mice, and, although this clearing is thought to represent oocyte culling (Lei and Spradling, 2013, 2016), the underlying mechanism remains unclear. In both humans and mice, the clearance of germ cells by apoptosis at least partly overlaps with the transition of oocytes from the cyst organization to forming the primordial and primary follicle. These developmental dynamics strongly suggest that germ cell clearance involves regulation in and/or by the germline cyst.

Supporting this notion, during oocyte culling the Bb in mice is thought to label oocytes for follicle formation (Lei and Spradling, 2016). It has been proposed that the Bb forms in oocytes that are fated to folliculogenesis, and does not form in oocytes fated to apoptosis (Lei and Spradling, 2016). Whether the Bb functionally promotes or simply marks oocyte development remains to be determined. However, in zebrafish and mice, the Bb begins to form in the cyst and matures in the primordial and primary follicles (Elkouby et al., 2016; Lei and Spradling, 2016). Therefore, these observations provide evidence for potential regulation of this crucial cell-fate decision in oogenesis through Bb formation and already in the cyst. An alternative, non-mutually exclusive, scenario has been suggested by recent observations in mice that some cyst cells are fated to become nurse cells, and only a few are fated as oocytes (Niu and Spradling, 2022). This mechanism was suggested to involve transfer of material, including Bb components, from nurse-like cells to the oocytes (Niu and Spradling, 2022), which further supports the potential contribution of Bb formation in the cyst to oocyte selection.

At birth, human ovaries contain a finite number of follicles. The convention is that germline stem cells are not maintained or produce oocytes *de novo* in the post-natal mammalian ovary (Lesch and Page, 2012). Recent reports suggest the existence of germline stem-like or mitotic germ cells in adult ovaries (Johnson et al., 2004; Eggan et al., 2006; Zou et al., 2009; Pacchiarotti et al., 2010; Kumar, 1968; White et al., 2012), but these remain controversial (Martin et al., 2019). Thus, the above events in the developing ovary, from oogonia to the primary follicle, are extremely crucial because they determine the number and quality of follicles for the entire lifespan of the person. Unfortunately, they are also the most challenging stages to address experimentally in humans, and we lack a fundamental understanding of these early processes and their developmental defects, which cause infertility, reproductive disease and malignancies.

The zebrafish executes developmental programs of oogenesis and gonad development that are conserved with mammals. The developmental stages, morphological processes and order of events of oocyte differentiation are similar (reviewed by Elkouby and Mullins, 2017a; Li and Ge, 2020), and so are many of the increasingly identified genetic regulators that control different facets of ovarian and oocyte development (Webster et al., 2017; Yang et al., 2017; Kossack and Draper, 2019; Qin et al., 2018; Beer and Draper, 2013; Cao et al., 2019; Houwing et al., 2007, 2008; Blokhina et al., 2021; Marlow and Mullins, 2008; Escobar-Aguirre et al., 2017a; Roovers et al., 2018; Chu et al., 2015; Zhang et al., 2015; Crowder et al., 2018; Yu et al., 2018).

Additional advantages of the zebrafish model include the high accessibility of developing ovaries that are present in swimming juvenile fish, and the flat and transparent anatomy of the ovary, which is ideal for advanced quantitative and live microscopy, as we demonstrated previously (Elkouby and Mullins, 2017a,b; Elkouby et al., 2016; Mytlis and Elkouby, 2021; Deis and Elkouby, 2022 preprint; Mytlis et al., 2022), and in this work. Furthermore, in zebrafish germline stem cells are maintained and actively produce oocytes throughout life and during regeneration (Beer and Draper, 2013; Cao, et al., 2019). Although the process of oogenesis is similar to that in mammals, the exception in zebrafish is its capacity to non-synchronously repeat the same process multiple times. As a result, all stages of early oogenesis can be abundantly found in the developing ovary, which enables a holistic view of these processes. In the mouse, oocytes differentiate synchronously, and at any given developmental time frame a predominant oocyte stage populates the developing ovary. In this sense, the fact that the zebrafish ovary non-synchronously contains mitotic oogonia adjacent to differentiating oocytes at various stages makes it more similar to the non-synchronous differentiation of oocytes in the developing human ovary (Kurilo, 1981; Farini and De Felici, 2022). Thus, the zebrafish ovary provides an excellent model for understanding the cellular mechanisms that control oogenesis and ovarian development. Specifically, it offers a promising model to identify the overlooked mechanisms that govern morphogenesis of the germline cyst, with direct relevance for human reproduction.

Altogether, the methods described here provide a new toolkit for analyzing germline cyst morphology, organization and development in early oogenesis in zebrafish. These methods harness cutting-edge tools and serve as a steppingstone to address fundamental long-sought-after questions in oogenesis, with implications generally in cell, developmental and reproduction biology. Furthermore, they can be transferred to other developmental systems in zebrafish, as well as other species, providing a means of comprehensive, unbiased and quantitative analysis.

## MATERIALS AND METHODS

### Ethics statement

All animal experiments were supervised by the Hebrew University Authority for Biological Models and were appropriately approved under ethics requests MD-2016222-1 and MD-18-15600-2.

### Fish lines and gonad collections

Juvenile ovaries were collected from 5–7 week post-fertilization (wpf) juvenile fish. Fish had a standard length (SL) measured according to Parichy et al. (2009), and were consistently ∼10-15 mm. Ovary collection was carried out as previously described (Elkouby and Mullins, 2017b). Fish lines used in this research were: TU wild type, *Tg(β-act:Arl13b-GFP)* (Borovina et al., 2010), *Tg(β-act:Cetn2-GFP)* (Novorol et al., 2013), *Tg(h2a:H2A-GFP)* (Pauls et al., 2001) and *Tg(β-act:LifeAct-GFP)* (Behrndt et al., 2013)*.*

### SBF-SEM data segmentation and volume reconstruction

Ovaries were dissected as previously described (Mytlis and Elkouby, 2021) and embedded in blocks as described (Mytlis et al., 2022). Images were acquired using a Gatan 3View serial block-face SEM system mounted on a Quanta 250 SEM (FEI). Imaging conditions were as follows: magnification 3400-3500×, pixel size 5.9 nm in *x* and *y*, 75 nm in *z*. Images were binned by two giving a final pixel size in *x* and *y* of 12 nm (Mytlis et al., 2022).

### Protocol for manual segmentation of SBF-SEM data using TrakEM2

1. Import Image files into Fiji or ImageJ as stack (Fig. 2A, left).
  File>Import>Image Sequence
2. Correct the imported image stack for brightness/contrast and alignment.
  Image>Adjust>Brightness/Contrast
3. Open a new TrakEM2 (blank) workspace.
  File>New>TrakEM2 (blank)
4. Select a directory for saving TrakEM2 temporary files.
5. In the TrakEM2 workspace panel, right-click on empty space, import the image stack.
6. While importing, in the Slice Separation dialog box, manually enter the voxel depth and check the box ‘One Slice Per Layer’.
7. In the TrakEM2 organizer panel, select the Template tab. Create a new ‘Area_list’ under ‘Add new child’.
8. Drag and drop the area_list from the Template tab to the Project Object tab.
9. In the Project Object tab, select the area_list and rename it to the intended structure to be segmented.
10. Select the area_list from step 8, under the ‘Z Space’ tab.
11. From the TrakEM2 workspace, select the ‘Brush Tool’ and the desired size.
12. Draw a contour on the structure of interest on alternate slices (Fig. 2A, middle).
13. Once all the slices are marked, fill the empty slices by interpolating the contours.
  Menu>Areas>Interpolate All Gaps
14. Repeat steps 7-13 for all the structures of interest.
15. Export the area_lists from the TrakEM2 workspace. Set the scale to 100.
  Menu>Export>Arealists as labels (tif)
16. Save the exported labels image to the local directory.
  File>Save as>Tiff

### Protocol for volume reconstruction in Imaris

1. From the Imaris homepage, open the working directory and import the labels in the Imaris Arena Tab.
2. Set the voxel size according to the raw image data.
  File>Image Preferences
3. In the Surpass Tree Item Menu, select the ‘Create New Surface’ tool from the Surpass Tree Item Menu.
4. In the Creation dialog box, uncheck the ‘Classify Surfaces’ and ‘Track Surface over time’ options.
5. Select ‘Absolute Intensity’ and select he area around the peak in the histogram to segment.
6. Set the desired color and transparency for the surface created in step 5 (Fig. 2A, right).
7. Repeat steps 3-6 for all surfaces of interest in the labeled image. Save the surface by exporting as ‘Scenes’.
8. Import the raw dataset in the Surpass workspace and the surfaces saved in step 7.
  File>Import Scenes
9. Add ‘Orthoslicer’ from the Surpass Tree Item Menu. Set the slice of choice.
10. Note: Uncheck ‘Volume’ in Surpass Tree Items to hide the raw dataset.
11. Go to the ‘3D Animation’ tab from the Surpass workspace menu.
12. Adjust the scene in the desired orientation.
13. Add the Animation option and the total number of frames.
14. Hit ‘Record’, and select the directory for saving the animation (Fig. 2B-G).

### Deep learning-assisted instance segmentation

Sample preparation for live time-lapse imaging and immunostaining was previously described (Elkouby and Mullins, 2017b; Mytlis and Elkouby, 2021; Mytlis et al., 2022). Images were acquired on a Zeiss LSM 880 confocal microscope using a 40× lens. The acquisition setting was set between samples and experiments to: *xy* resolution=1104×1104 pixels, 12-bit, 2× sampling averaging, pixel dwell time=0.59 s, zoom=0.8×, pinhole adjusted to 1.1 μm of *z* thickness, increments between images in stacks 0.53 μm, laser power and gain set in an antibody-dependent manner to 7-11% and 400-650, respectively, and below saturation conditions.

#### Protocol for setting up the working environment

1. Install Anaconda environment manager (https://www.anaconda.com/).
2. Create a virtual environment in Anaconda.
3. Install Jupyter Notebook.
4. Download the Jupyter notebooks for StarDist (https://github.com/stardist/stardist.git) and Cellpose (https://github.com/MouseLand/cellpose.git) from the respective GitHub repositories.
5. Launch the Anaconda Terminal.

#### Protocol for preparing the training dataset for StarDist

1. Open the images in Fiji. Using the crop tool, crop regions of 256×256 or 128×128.
2. Save the Crop regions in a directory named ‘Training Images’.
3. Open Training Images in Fiji and annotate all the structures of interest using Labkit (Arzt et al., 2022) or TrakEM2 (Cardona et al., 2012).
4. Export and save the labeled images in a directory named ‘Training Mask’.

#### Protocol for model training and predictions

1. From the Anaconda Terminal, activate the respective environment.
2. Launch Jupyter Notebook and browse to the Jupyter notebook downloaded earlier.
3. In the Notebook, enter the path to ‘Training Images’ and ‘Training Mask’ directories in the respective fields.
4. Enter the model name and the directory path in which to save it.
5. Train the model until the training curve plateaus.
6. Evaluate the quality of the model by looking at:
  - Inspection of loss function. The validation loss and training loss curves should converge at the end of training for successful model training. If the validation loss increases with the decrease of training loss, the model is overfitting and the training dataset should be increased.
  - ‘Intersection of Union (IOU)’. The closer to 1, the better the performance. (If IOU is less, the training dataset should be increased.)

Once the model is trained it can be further used to make predictions on unseen datasets.

7. Enter the path to unseen datasets in Jupyter Notebook (Fig. 3B). Choose the Custom Model.
8. Run the program to make predictions on the unseen dataset using the above created model (Fig. 3C).

#### Protocol for cell segmentation using Cellpose

1. Launch a new Jupyter browser in the Cellpose environment using the Anaconda environment manager.
2. Open a Cellpose notebook in the Jupyter notebook.
3. Provide the directory path for images to be predicted (Fig. 3B) and save results.
4. Choose the provided model.
5. Set do_3d=True for segmentation done using 3D image or set do_3d=False for 2D segmentation and stitching of labels.
6. Set the minimum diameter of cell in pixels, if do_3d=True in step 5.
7. Proceed to segmentation.

#### Protocol for features extraction from label images

1. Import the predicted label images into Fiji.
2. Set the voxel size to raw data voxel size from properties.
  Fiji>Image>Properties
3. Correct the labels for misidentification using the label editor from the MorphoLibJ plugin (Legland et al., 2016).
4. Extract the features from the 3D label images using plugin ‘Analyze Regions 3D’ from MorphoLibJ (Legland et al., 2016) (Fig. 3E).
5. Set the Glasbey colormap LUT on the predicted label image from LUT menu.
  Fiji>Image>Lookup Tables>Glasbey on Dark
6. Open 3D viewer from the Fiji Plugins menu.
  Fiji>Plugins>3D viewer
7. Select the filename from the drop-down menu in the import dialog box. Import as Volume.
8. Visualize the spatial arrangement of segmented structures in volume reconstruction.
9. Volume reconstruction can also be generated by importing the label images in to the Imaris Workspace followed by creating a surface using the ‘Surface Creation Tool’ (Fig. 3D).

### Laser-induced ablation of cyst cell organelles

#### Ovary mounting and culture

Ovaries were isolated as described previously (Elkouby and Mullins, 2017b; Mytlis et al., 2022) (Fig. 4A1,2). A dissecting dish [made in-house by casting plastic Petri dishes with animal-proof nontoxic silicone for reusable dishes or 2-3% agarose in Hank's solution (0.137 M NaCl, 5.4 mM KCl, 0.25 mM Na_2_HPO_4_, 0.44 mM KH_2_PO_4_, 1.3 mM CaCl_2_, 1.0 mM MgSO_4_, 4.2 mM NaHCO_3_) for single-use dishes], micro-scissors and Forceps #5 (Sigma-Aldrich) were used for ovary dissection (for details, see Mytlis and Elkouby (2021). Dissected ovaries were kept in a glass 9-well plate at 28°C until mounting.

In a glass-bottom dish (60 µ, ibidi), ∼150 µl of mounting solution was added (agarose layer 1) and left until it started to solidify. Ovaries were transferred carefully to the mounting solution (agarose layer 1) in the glass-bottom dish using forceps (Fig. 4A3). The ovaries were gently pushed to the bottom of the dish, avoiding curls, as described previously (Elkouby and Mullins, 2017b; Mytlis et al., 2022), and allowed to rest until the agar solidified (Fig. 4A3). Once the agar had solidified, more mounting solution was added (agarose layer 2) until it covered the solidified mounting solution containing the ovaries (Fig. 4A3), and then allowed to rest for the agar to solidify properly. An adequate volume (∼1.5 ml) of HL-15 medium (60% Hanks, 40% L-15, 1:100 GlutaMAX) was added to the cell culture dish (Fig. 4A3). Note that HL-15 should be stored at 4°C. L-15 (2× stock) was used without L-glutamine and Phenol Red. L-glutamine is not stable and should be added fresh from a stock (GlutaMAX 100×; Gibco, 35050-061; store at room temperature). Mounted ovaries were kept at 28°C until use. Agarose layers 1 and 2 were prepared by mixing 500 µl of Mounting Solution A (1% low-melt agarose in Hank's Solution; store at 4°C) with 500 µl of Mounting Solution B [490 µl of 2× L-15 (no L-glutamine, no Phenol Red) and 10 µl GlutaMax; equivalent to a 2× HL-15 solution; make fresh and keep at 28°C] to make a final solution of 0.5% low-melt agarose in 1× HL-15 (gelling temperature, 27.4°C).

#### Laser-induced ablation

Laser excisions were performed using a Leica TCS SP8 MP two-photon microscope with a 25× objective and equipped with an incubation chamber set to 28°C. The glass-bottom dish with the cultured ovaries was mounted on the microscope stage inside the incubator chamber. The region of interest (ROI) was located using a 25× objective. The desired zoomed view of the ROI was obtained using ‘Digital Zoom’ and ‘Capture a Live View’. and draw The ROI for ablation was drawn on the above acquired image using ‘ROI tools’. Once the ROI was marked, the imaging time parameters were set as follows: pre-ablation timelapse acquisition 60 s; laser stimulation of the ROI 60 s at laser power 2.0-8.0% out of a power source of ∼3 W; post-ablation timelapse acquisition 600 s. These steps were repeated for each ROI. Note that only one ablation per cyst should be performed to avoid cell and tissue damage. Ovaries (4-6 wpf) should be mounted in the cell culture dish towards the center, leaving enough space for the lens to move around.

#### Software

Fiji was used for the preprocessing of image datasets and post processing of labeled images. Anaconda, an open-source distribution of Python was used specifically to maintain a dedicated virtual environment with the desired versions of Python packages installed. Jupyter Notebook, a web-based interactive computational environment for creating and sharing documents, was used to run deep-learning algorithms. Cellpose (Stringer et al., 2021) is an anatomical segmentation algorithm written in Python3. StarDist (Weigert et al., 2020; Schmidt et al., 2018) is a deep learning based-algorithm for star-convex object detection for 2D and 3D images. Imaris is a commercial microscopy image analysis software.

## Supplementary Material

10.1242/develop.201349_sup1 Supplementary information
