# Supplementary material for: Tools to analyze the organization and formation of the germline cyst in zebrafish oogenesis
Source: Development. 2023 Jun 30;150(13):dev201349. doi: 10.1242/dev.201349 (PMC10323243; doi:10.1242/dev.201349)
Supplement: Supplementary information [file develop-150-201349-s1.pdf]

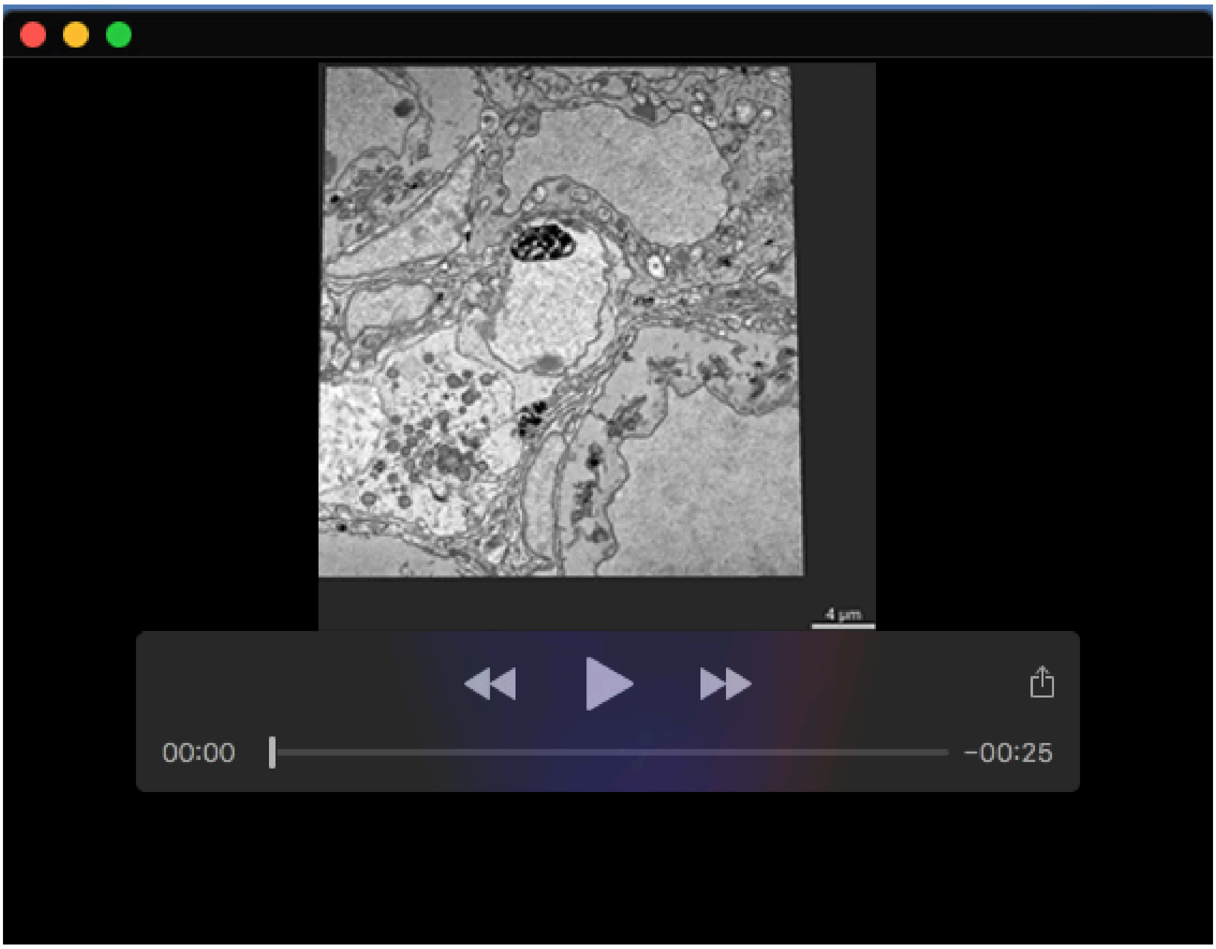

Movie 1. Volume Reconstruction of zygotene cyst cells by manual segmentation of three dimensional SBF-SEM imaging data, as shown in Fig. 2. Cytoplasmic membranes (brown and pink), centrosome (green), zygotene cilium (maroon), mitochondria (beige), and nuclei grey are shown.

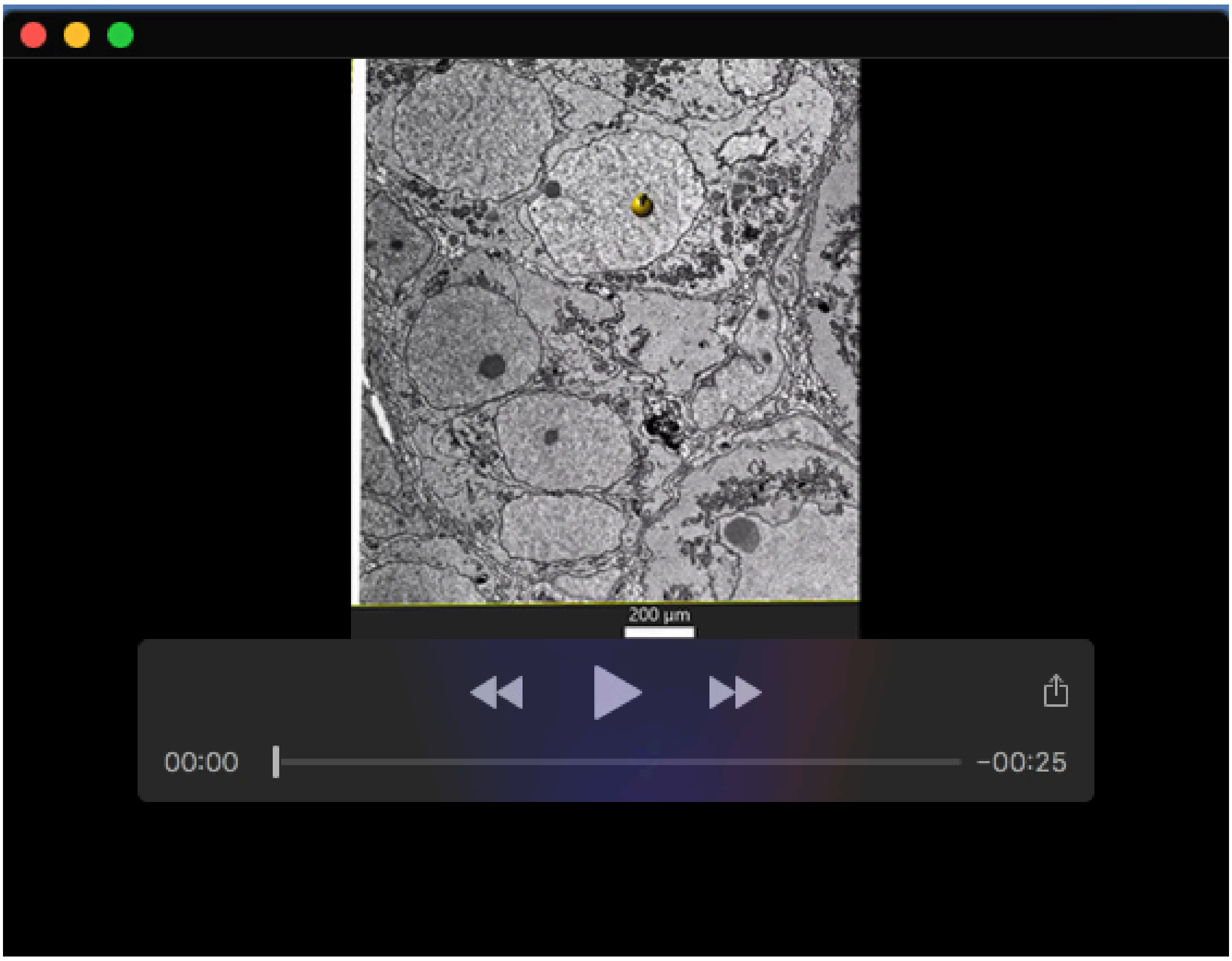

Movie 2. Volume Reconstruction of leptotene cyst cells and their CBs by manual segmentation of three dimensional SBF-SEM imaging data. Cytoplasmic membranes are color coded, CBs are in red.

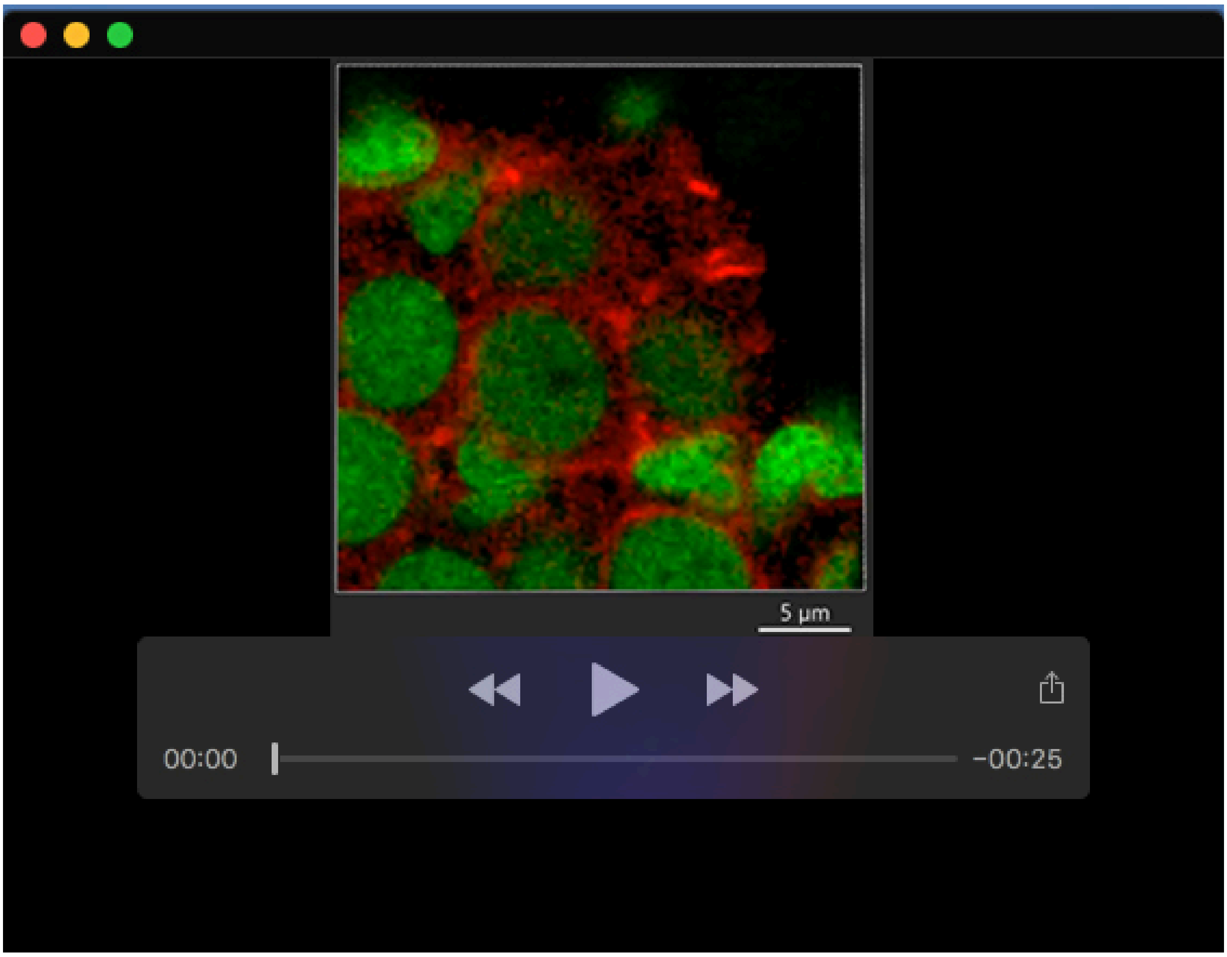

Movie 3. Volume reconstruction of Oogonia cyst using label images generated by instance segmentation deep-learning algorithms, as shown in Fig. 3A-D.

Development • Supplementary information

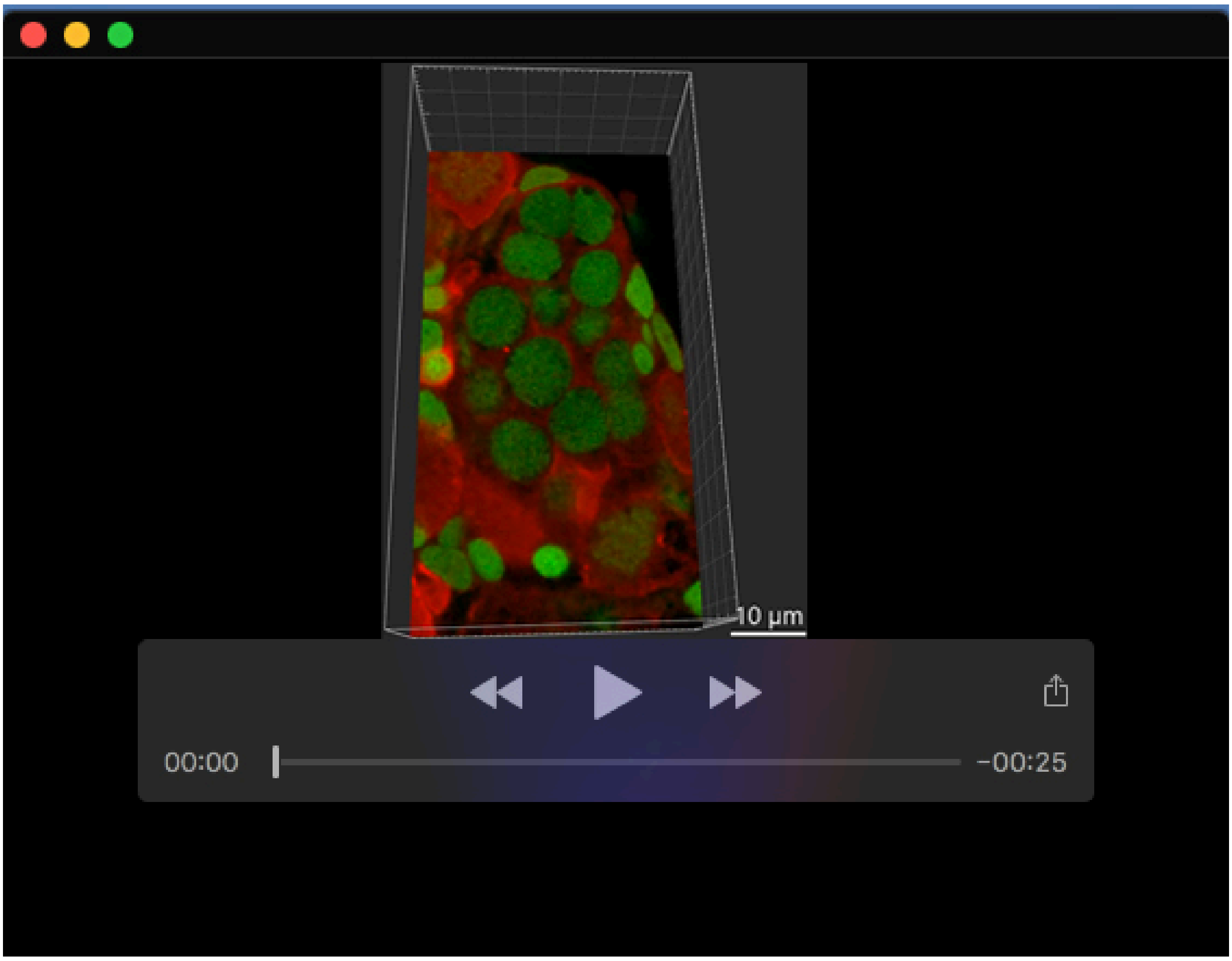

Movie 4. Volume reconstruction of Zygotene cyst using label images generated by instance segmentation deep-learning algorithms, as shown in Fig. 3A-D.

Development • Supplementary information

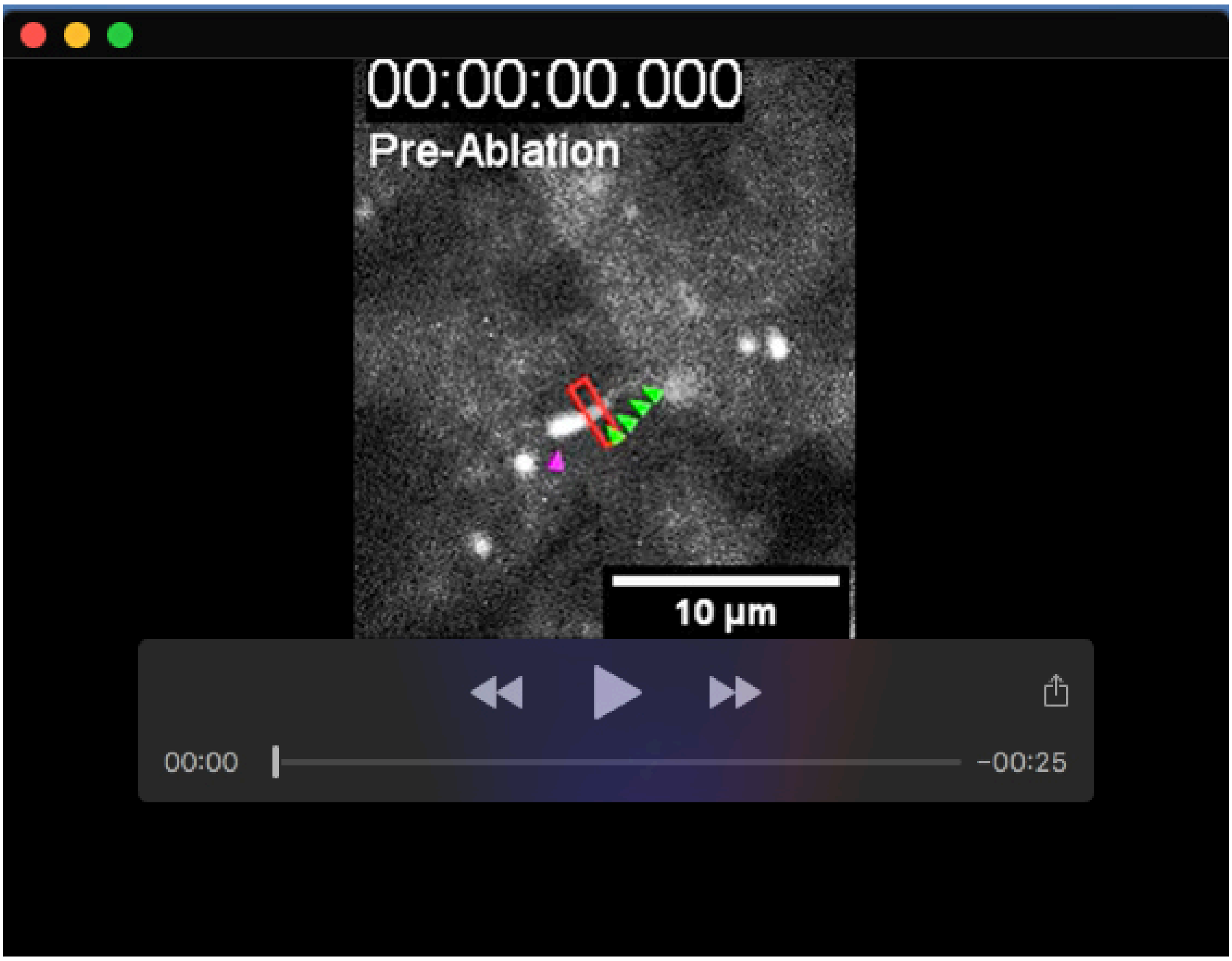

Movie 5. Live time-lapse recordings of the laser induced excision of the zygotene cilium shown in Fig. 4B.

Development • Supplementary information

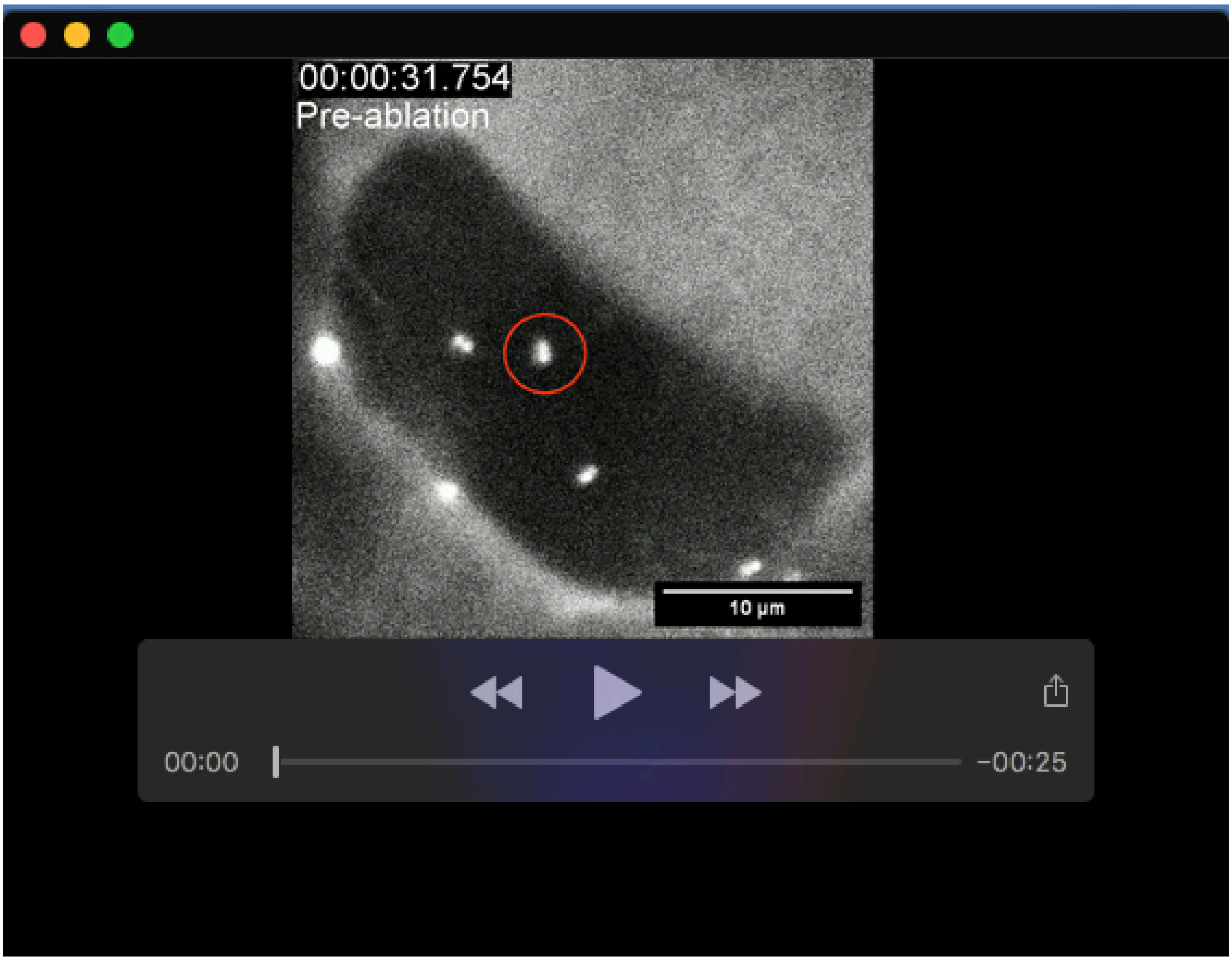

Movie 6. Live time-lapse recordings of the laser induced ablation of a single centrosome shown in Fig. 4C.

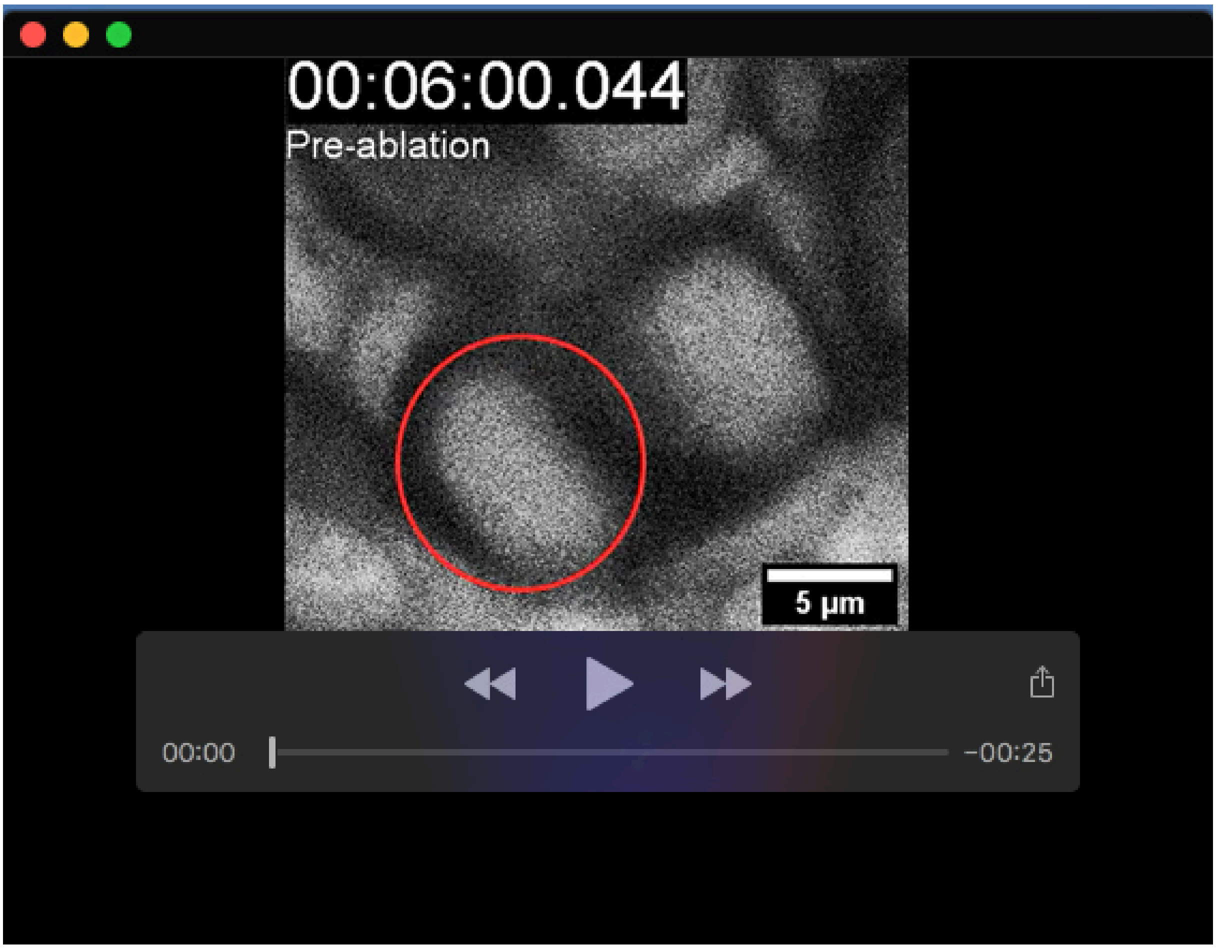

Movie 7. Live time-lapse recordings of the laser induced ablation of a single nucleus in a 2-cell oogonia cyst shown in Fig. 4D.

Development • Supplementary information

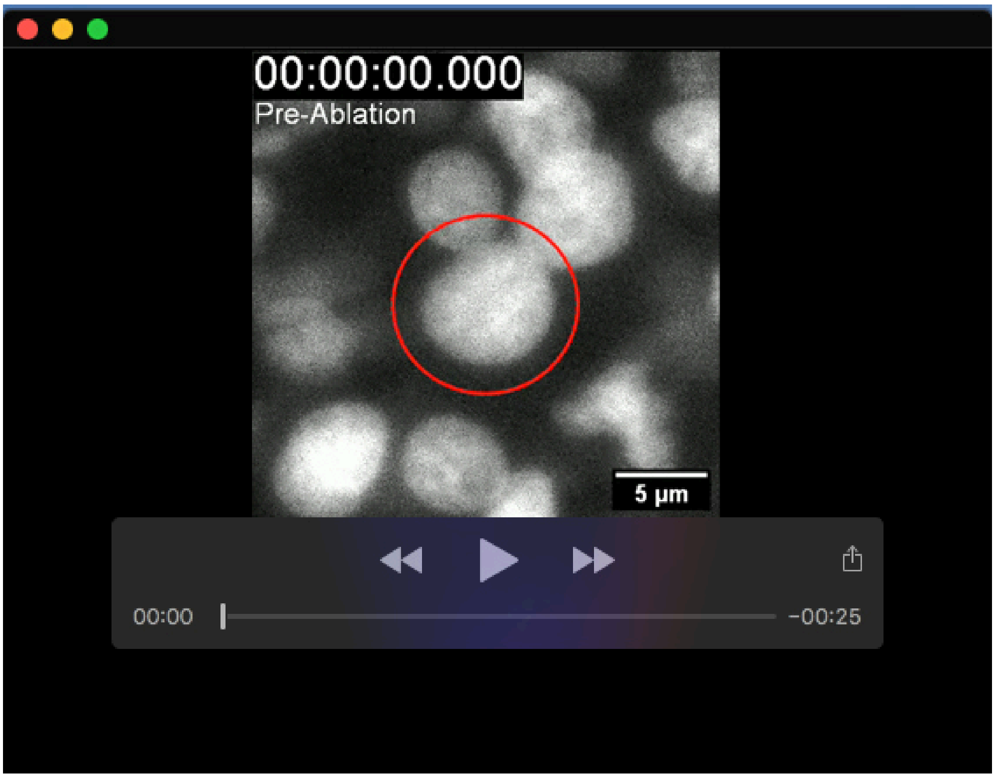

Movie 8. Live time-lapse recordings of the laser induced ablation of a single nucleus in meiotic leptotene and zygotene cysts shown in Fig. 4E.

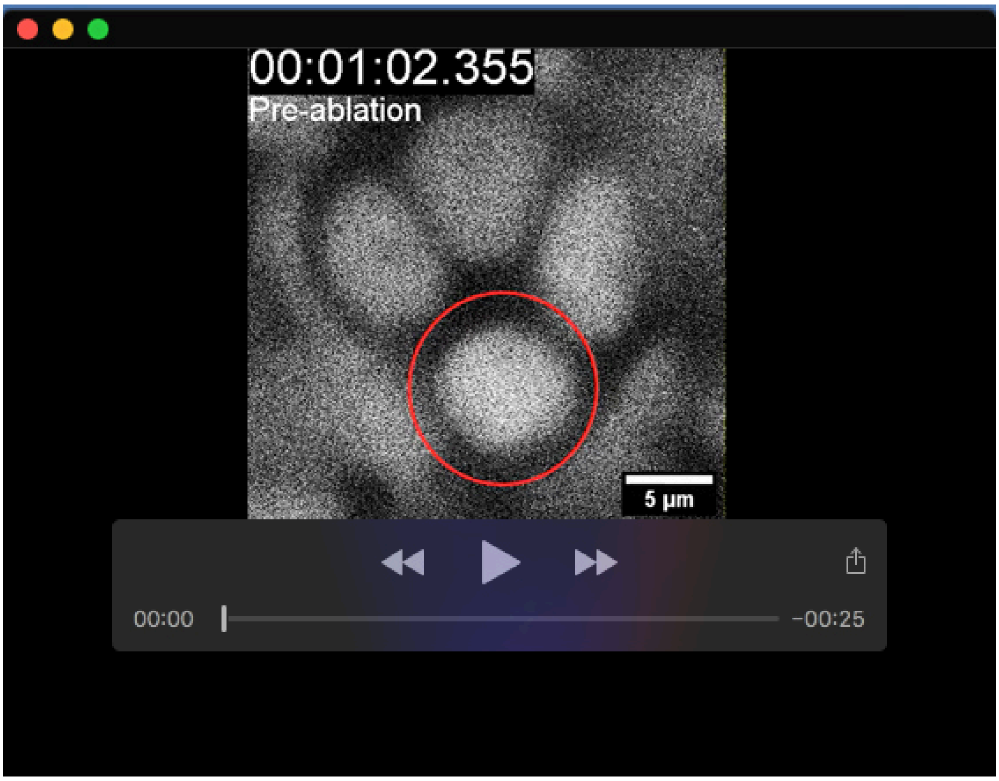

Movie 9. Live time-lapse recordings of the laser induced ablation of a single nucleus in meiotic leptotene and zygotene cysts shown in Fig. 4E.

Development • Supplementary information
